# Multi-drug-resistant *Enterococcus faecium* bacteraemia in a liver transplant recipient

**DOI:** 10.1099/jmmcr.0.005172

**Published:** 2018-12-20

**Authors:** Nathan A. Summers, John Gharbin, Rachel Friedman-Moraco, G. Marshall Lyon, Joseph Lutgring

**Affiliations:** ^1^​ Division of Infectious Diseases, Department of Medicine, Emory University, Atlanta, GA, USA; ^2^​ Department of Global Health, Emory University, Atlanta, GA, USA

**Keywords:** bacteraemia, liver abscess, chloramphenicol, tigecycline, solid organ transplant

## Abstract

**Introduction:**

*Enterococcus faecium* is a commensal organism commonly colonizing the human gastrointestinal tract. Although it is generally a non-virulent organism, *E. faecium* can cause significant morbidity and mortality due to its inherent and acquired resistances to commonly used antimicrobials. Patients who are immunosuppressed are particularly vulnerable.

**Case presentation:**

A 65–75-year-old patient with a history of an orthotopic liver transplant for hepatitis C infection and diabetes was re-admitted to the hospital with abdominal pain and fever. The patient had several recent admissions related to the presentation reported here, which included treatment with a prolonged course of broad-spectrum antibiotics. The patient was found to have a recurrent liver abscess and blood cultures grew vancomycin-resistant *E. faecium*, non-susceptible to all tested agents: ampicillin, penicillin, vancomycin, daptomycin and linezolid. The patient was started initially on chloramphenicol intravenously while awaiting additional susceptibility testing, which ultimately revealed chloramphenicol non-susceptibility. Tigecycline was started but the patient ultimately decided to pursue hospice care.

**Conclusion:**

Multi-drug-resistant organisms are increasingly being recognized and are associated with poorer outcomes, particularly in immunosuppressed patients. We describe a particularly resistant organism and discuss potential therapeutic options.

## Introduction

*Enterococcus* species, including *Enterococcus faecium*, are frequent colonizers of the human gastrointestinal tract and are frequently viewed as commensal organisms [1]. Although not typically considered to be virulent organisms, *Enterococcus* species, particularly *E. faecium*, can have significant pathogenic potential due to both inherent and acquired resistances to many commonly used antibiotics used today [1, 2]. Despite advances in clinical care, including newer antimicrobials, outcomes for infections caused by *Enterococcus* species are poor, and are considerably worse for vancomycin-resistant *E. faecium* (VRE) compared to susceptible strains [2]. Multi-drug-resistant organisms are increasingly recognized as problems for immunocompromised patients, particularly VRE [3]. Having a deeper understanding of the antimicrobials available, as well as their potential toxicities and drug–drug interactions, is vitally important to improving patient outcomes.

## Case report

Our patient was a 65–75-year-old with a history of orthotopic liver transplant in 2000 for hepatitis C infection, on chronic immunosuppression with cyclosporine, pancreatic adenocarcinoma status post-Whipple procedure in 2015, and diabetes mellitus, who was admitted for fever and worsening abdominal pain. The patient had several recent admissions in the preceding 3 months for perihepatic abscess and bacteraemia. Three months prior to the admission reported here, liver abscess cultures obtained during drain placement grew *Rothia mucilaginosa* and α-haemolytic *Streptococcus* species, with *Streptococcus parasanguinis* growing in two sets of blood cultures. The patient was sent home on ceftriaxone 2 grams (gm) intravenously (IV) every 24 h and metronidazole 500 milligrams (mg) by mouth (PO) every 8 h but was readmitted 1 month later with *Candida parapsilosis* fungaemia, felt to be related to the peripherally inserted central catheter (PICC). The PICC was removed and antimicrobials were changed to moxifloxacin 400 mg PO once daily and a 2 week course of fluconazole 400 mg PO every 24 h. The patient was readmitted 1 month before the admission reported here while still on the moxifloxacin, and was found to have worsening abdominal pain and an enlarging perihepatic abscess on a computerized tomography (CT) scan of the abdomen. A new drain was placed into the liver abscess, and cultures from this drain grew VRE. Antibiotics were then adjusted to daptomycin (dosed at 10 mg kg^−1^ every 24 h) and moxifloxacin 400 mg PO every 24 h for presumed polymicrobial infection, and the patient was discharged with a new PICC to complete a tentative 4–6-week course.

**Fig. 1.**
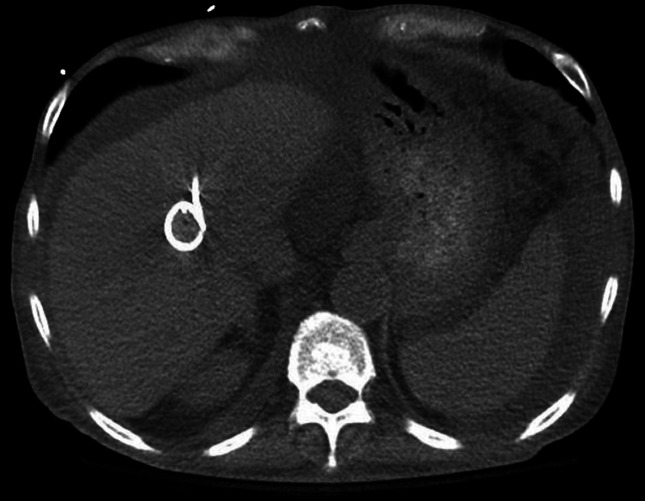
CT scan of the liver abscess with the drain in place.

At the beginning of the patient’s admission reported here, approximately 2 weeks after the most recent discharge, the antibiotics were changed to daptomycin 10 mg/kg every 24 h and meropenem 500 mg every 8 h (adjusted for his renal impairment) for improved Gram-negative and anaerobic coverage. A CT scan of the patient’s abdomen and pelvis was obtained (Fig. 1). Blood cultures returned with VRE, non-susceptible to ampicillin, penicillin, vancomycin, daptomycin and linezolid on initial susceptibilities performed by the MicroScan WalkAway-*96 plus* system with the Pos Combo 33 panel (Beckman Coulter Diagnostics). The daptomycin MIC was confirmed to be 8 µg ml^−1^ with ETEST (bioMérieux).

Additional susceptibilities were requested for chloramphenicol and tigecycline by ETEST. Susceptibility testing for quinupristin/dalfopristin, telavancin, dalbavancin and oritavancin was not performed because these agents were not on formulary and access to these therapeutics was not possible at the time. Quinupristin/dalfopristin was not used because it interacts with calcineurin inhibitors leading to supratherapeutic drug levels of cyclosporine and tacrolimus. The patient’s PICC was removed and the patient was started on intravenous chloramphenicol while awaiting further workup. This decision was based on the patient’s resistance pattern, as well as greater serum concentrations of chloramphenicol compared to tigecycline. Additional testing revealed non-susceptibility to chloramphenicol (MIC=16 µg ml^−1^). The isolate was susceptible to tigecycline (MIC ≤ 0.25 µg ml^−1^), so the decision was made to transition to tigecycline monotherapy, as it was felt that adequate serum concentrations were achievable with such a low MIC. The patient initially tolerated this change well and was discharged home with a new PICC. Unfortunately, the patient was later admitted with persistent failure to thrive including nausea, diarrhoea and weakness 1 month later. Palliative care was considered and the patient decided to pursue hospice care, declining further antimicrobial treatment.

## Discussion

Multi-drug-resistant *E. faecium* treatment is an increasing challenge, confronting clinicians globally. Although current recommendations encourage the use of daptomycin or linezolid as the first-line treatment options for VRE [4, 5], data is lacking for solid organ transplant recipients on immunosuppressive agents or in cases where the first-line antimicrobials are ineffective. Alternative therapeutic agents for the treatment of VRE in the presence of resistance to ampicillin, penicillin, vancomycin, daptomycin and linezolid are limited. Quinupristin/dalfopristin is active against *E. faecium* but not *Enterococcus faecalis*, but it is poorly tolerated, requires central venous access for administration, and increases serum concentrations of calcineurin inhibitors and mTOR inhibitors, which are commonly used in solid organ transplant recipients [3]. Tigecycline also possesses activity against VRE, but achieves very low serum concentrations and there is concern for increased mortality compared to other agents [6]. Telavancin, a lipoglycopeptide, is active against VRE strains possessing *van* B but not *van* A. Oritavancin, a long-acting lipoglycopeptide, is active against VRE strains possessing both *van* A and *van* B [7, 8], but clinical data for VRE bacteraemia is sparse.

Chloramphenicol possesses activity against VRE, but its toxicities limit widespread use today. Chloramphenicol is associated with both a reversible, dose-dependent bone marrow suppression, as well as dose-independent, irreversible aplastic anaemia [9, 10]. Although the mechanisms are not fully clear, the irreversible aplastic anaemia is primarily seen with oral administration of the agent, but not with intravenous administration. It is felt that enteric bacteria may play a role by degrading chloramphenicol, releasing toxic metabolites that are then absorbed enterally. The *p*-nitrosulfathiazole group, which inhibits DNA synthesis, is believed to be the causative metabolite, supported by the fact that thiamphenicol, available in Europe but not currently in the USA, does not possess this group and is not associated with aplastic anaemia [9, 10].

It was surprising to find chloramphenicol resistance despite a lack of prior exposure to the agent. Chloramphenicol resistance has been described previously and appears to be related to recent exposure to fluoroquinolones, as was the case for our patient [11]. Resistance is typically mediated through a multi-drug efflux pump, conveying resistance to fluoroquinolones, tetracyclines and chloramphenicol [12]. It is likely that our patient’s recent prolonged exposure to moxifloxacin upregulated this efflux pump, conveying resistance to chloramphenicol despite the patient’s lack of prior exposure.

Multi-drug resistant and extensively drug resistant organisms are becoming more common with the increased use of broad-spectrum antimicrobials, especially in the immunocompromised host [3]. As resistance develops to the preferred agents, it is imperative to understand the strengths and limitations of alternative agents, along with emerging resistance mechanisms.
